# Evaluation of the Anticonvulsant Effect of Brilliant Blue G, a Selective P2X7 Receptor Antagonist, in the *iv* PTZ-, Maximal Electroshock-, and 6 Hz-Induced Seizure Tests in Mice

**DOI:** 10.1007/s11064-017-2348-z

**Published:** 2017-07-12

**Authors:** Dorota Nieoczym, Katarzyna Socała, Piotr Wlaź

**Affiliations:** Faculty of Biology and Biotechnology, Department of Animal Physiology, Institute of Biology and Biochemistry, Maria Curie-Skłodowska University, Akademicka 19, 20-033 Lublin, Poland

**Keywords:** P2X7 receptor, Brilliant blue G, Acute seizure models, Mice

## Abstract

Epilepsy is one of the most common neurological disorders which is diagnosed in around 65 million people worldwide. Clinically available antiepileptic drugs fail to control epileptic activity in about 30% of patients and they are merely symptomatic treatments and cannot cure or prevent epilepsy. There remains a need for searching new therapeutic strategies for epileptic disorders. The P2X7 receptor has been recently investigated as a new target in epilepsy treatment. Preclinical studies revealed that P2X7 receptor antagonists have anticonvulsant properties in some models of epilepsy. We aimed to investigate whether P2X7 receptor antagonist—brilliant blue G (BBG)—is able to change seizure threshold in three acute seizure models in mice, i.e., in the intravenous pentylenetetrazole seizure threshold, maximal electroshock seizure threshold and 6 Hz psychomotor seizure threshold tests. BBG was administered acutely (50–200 mg/kg, 30 min before the tests) and sub-chronically (25–100 mg/kg, once daily for seven consecutive days). Moreover, the chimney and grip strength tests were used to estimate the influence of BBG on the motor coordination and muscular strength in mice, respectively. Our results revealed only a week anticonvulsant potential of the studied P2X7 receptor antagonist because it showed anticonvulsant action only in the 6 Hz seizure test, both after acute and sub-chronic administration. BBG did not significantly influence seizure thresholds in the remaining tests. Motor coordination and muscular strength were not affected by the studied P2X7 receptor antagonist. In summary, BBG does not possess any remarkable anticonvulsant potential in acute seizure models in mice.

## Introduction

Epilepsy is a common and chronic disorder experienced by about 65 million people across the globe. It is characterized as recurrent unprovoked seizures which are transient signs and/or symptoms of abnormal excessive and/or synchronous action of neurons in the brain [1]. Despite the fact that numerous antiepileptic drugs (AEDs) have been introduced in past decades, about one-third of affected population remains refractory to current treatment, and many others experience severe side effects such as sedation and/or cognitive dysfunction. Moreover, all available AEDs enable only symptomatic treatment and they cannot cure or prevent the disease. Therefore there is still an unfulfilled need to identify new agents with a better side effects profile to control drug-resistant seizures and to prevent or impair process of epileptogenesis [2, 3].

Until recently, synaptic dysfunctions and properties of neuronal membrane were analyzed as principal epilepsy mechanisms and therapeutic strategy. However, several novel approaches to epilepsy treatment are investigated currently and particular attention is paid to neuron–glia interactions and purinergic signaling. Purinergic neurotransmission includes receptors for adenosine and adenosine 5′-triphosphate (ATP) classified as P1 and P2, respectively. The P1 receptors are also called adenosine receptors (ARs) and four kinds of these are defined—A1, A2A, A2B and A3. Among P2 receptors two subfamilies are distinguished, i.e., ionotropic P2X receptors (mammalian P2X1–7), which are involved in fast synaptic transmission and synaptic plasticity, and metabotropic P2Y receptors (mammalian P2Y1, 2, 4, 6, 11, 12, 13, 14) which typically mediate slow responses to nucleotides. Although purinergic receptors are widely expressed through the central nervous system both in neurons and glia cells, P2X2 and P2X4 receptors are thought to be characteristic for neurons while P2X1/5 and P2X7 receptors dominate in astrocytes. P2X7 receptors, which are of interest to the present study, are involved in glia–glia and neuron–glia communication [4–7].

Interest has developed in the role of P2X7 receptors in the pathophysiology of numerous neurological disorders [8, 9], i.e., epilepsy [5, 6], Huntington’s disease [4], Alzheimer’s disease [10], migraine [11], pain [12] and mood disorders [13, 14]. These receptors are membrane-bound, non-selective cation channels, which are activated by high extracellular ATP concentration. Compared to other P2X receptors, P2X7 receptor has the lowest affinity for ATP, it needs mM levels for activation [15]. The characteristic feature of these receptors is also their slow desensitization. The P2X7 receptor allows rapid and nonselective passage of Na^+^, K^+^ and Ca^2+^ across the cell membrane, and what is more, their prolonged activation by agonist leads to formation of reversible plasma membrane pore permeable to larger molecular weight (up to 800 Da) molecules. They are expressed in neurons both pre- and postsynaptically as well as in glia (i.e., microglia cells, astrocytes, oligodendrocytes, Schwann cells) in various regions of central nervous system, i.e., in cortex, striatum, brainstem, nucleus accumbens, cerebellum and hippocampus [5, 6, 15]. Wide distribution of P2X7 receptors across the central nervous system enables them to modulate variety of signaling pathways. First of all, trans-membrane fluxes of Na^+^, K^+^ and Ca^2+^ caused by P2X7 receptor pore formation mediate fast excitatory neurotransmission and modulate release of neurotransmitters (i.e., glutamate, GABA, ATP) both from neuronal terminals and astrocytes. These receptors might be also involved in the cross-talk between glial and neuronal cells. Moreover, the P2X7 receptors mediate cellular processes such as cell proliferation, apoptosis and inflammatory processes [5, 6, 9, 15].

In addition to dysfunctions of neurotransmission, neuroinflammatory processes seem also to be an important factor in pathogenesis of epilepsy. Numerous experimental and clinical results showed increased level of pro-inflammatory factors in brain tissues both from human epileptic patients [16] and animal models [17, 18]. Since P2X7 receptors have relatively scant affinity to ATP they are mainly activated under conditions of pathologically high concentration of ATP elicited in the wake of brain injury, ischemia or pathologic brain activity, such as during prolonged and repeated seizures. Therefore, ATP might be considered as a danger molecule which mediates pathological changes. Activation of P2X7 receptors promotes neuroinflammation and release of proconvulsive inflammatory cytokine interleukin 1β and tumor necrosis factor alpha [6, 19, 20].

Although contribution of P2X7 receptor in epileptogenic processes was widely investigated, little is known about influence of P2X7 receptor antagonists on seizure thresholds in experimental models of seizures. This fact inspired us to investigate the effect of brilliant blue G (BBG), a blood–brain barrier permeable selective P2X7 receptor antagonist [21], on seizure thresholds in three acute seizure models, i.e., in the 6 Hz seizure threshold, maximal electroshock seizure threshold (MEST) and intravenous pentylenetetrazole (*iv* PTZ) seizure threshold tests in mice. The potential anticonvulsant effect of BBG was investigated both after its acute and sub-chronic (7-day) administration. Moreover, some acute side effects provoked by BBG were examined in the chimney test (estimate motor coordination) and grip-strength test (estimate muscular strength) in mice.

## Materials and Methods

### Animals

Naïve male Swiss mice weighing 23–28 g obtained from a licensed breeder (Laboratory Animals Breeding, Słaboszów, Poland) were used in the study. The animals were housed in the polycarbonate cages under strictly controlled conditions (ambient temperature 20–23 °C, relative humidity 45–55%, a 12/12 light/dark cycle with the light on at 6:00 a.m., chow pellets and tap water continuously available). Mice were used in the study after at least one week of acclimatization. All experiments were performed at the same time of day (between 8:00 a.m. and 3:00 p.m.) to minimize circadian influences. Control and drug experiments were always done on the same day to avoid day-to-day variations in convulsive susceptibility. The total number of animals used in the present study was 582. All procedures were conducted in accordance with the European Union Directive of 22 September 2010 (2010/63/EU) and Polish legislation acts concerning animal experimentations. The experimental procedures and protocols were approved by the First Local Ethics Committee at the Medical University of Lublin (25/2014).

### Drugs

BBG (Sigma-Aldrich, Germany) was dissolved in saline and injected intraperitoneally (*ip*) at a volume of 0.1 ml per 10 g body weight. The studied compound was administered acutely (30 min prior to the tests) or sub-chronically (once daily for seven consecutive days) and the tests were performed 30 min after the last injection. The pretreatment time was selected during initial experiment which examined time-course effect of BBG in the 6 Hz seizure threshold test in mice. In order to verify the reliability of the seizure tests we additionally evaluated the influence of VPA (at a dose of 150 mg/kg in the *iv* PTZ and MEST tests and at a dose of 50 mg/kg in the 6 Hz test) on seizure thresholds in these tests (as positive control). VPA (as sodium salt; Sigma-Aldrich Co., St. Louis, MO, USA) was dissolved in saline and administered *ip* 15 min before the tests (acute treatment). All drug solutions were prepared freshly. Control animals received saline at the appropriate volume and time.

### The 6 Hz Electroshock-Induced Seizures

Psychomotor seizures (6 Hz seizures) were induced via corneal stimulation (0.2 ms square pulse at 6 Hz for 3 s) using Grass S48 stimulator coupled with a constant current unit CCU1 (both from Grass Technologies, West Warwick, RI, USA). A drop of ocular anesthetic, 1% solution of tetracaine hydrochloride (Sigma-Aldrich Co., St. Louis, MO, USA), was placed on the animals’ corneas before the stimulation and the electrodes were soaked in the 0.9% saline immediately before testing to ensure a good electrical contact. Mice were restrained manually during stimulation and placed in a Plexiglas box (35 × 20 × 14 cm) for observation immediately after stimulation. Six Hertz electroshock-induced seizures were characterized by stun, which was often followed by rearing, forelimb clonus, and twitching of vibrissae, Straub-tail, which lasted at least 10 s from the stimulation [22]. The animal was considered to be protected if it resumed its normal exploratory behavior within 10 s from the stimulation.

In order to determine the time course of anticonvulsant effect of BBG in the 6 Hz test in mice, the compound was injected *ip* at a fixed dose of 200 mg/kg and tested at 15, 30, 60, 120 and 240 min post-injection time points. We used a relatively high dose of the studied drug, i.e., 200 mg/kg, to detect its potential anticonvulsant effect. To evaluate effect of acute treatment with BBG animals (groups of 18–20 animals) were given with different doses (50–200 mg/kg) of this compound and tested 30 min after injection (at time point in which the studied compound was at most efficacious). In case of sub-chronic treatment, animals received BBG once daily for seven consecutive days at doses of 25–100 mg/kg. In both cases, control groups were treated with saline.

The mice were subjected to stimuli with different current intensities according to the “up-and-down” method and the median current strength (CS_50_ in mA; the current strength of 6 Hz stimulation which induce psychomotor seizures in 50% of the tested animals) with 95% confidence limits was calculated as described elsewhere [23, 24]. Each mouse was stimulated only once at any given current intensity (8–23 mA) and convulsant activity was judged as described above. If the mice responded with seizures, the next mouse was stimulated with current of an intensity 0.06-log step lower than the previous one. If the mice did not have seizures, the next one was stimulated with a current of intensity 0.06-log step higher [23].

### The Maximal Electroshock Seizure Threshold Test

The seizures were induced by applying a sine-wave alternating current (maximal output voltage 500 V, 50 Hz for 0.2 s) via transcorneal copper electrodes. A constant current stimulator (Rodent Shocker, Type 221, Hugo Sachs Elektronik, Freiburg, Germany) was used. Ocular anesthetic (1% solution of tetracaine hydrochloride) was applied to animals’ eyes before stimulation and 0.9% saline was used to wet electrodes before testing to provide a good electrical output. Animals were manually restrained during stimulation and immediately after stimulation they were placed in a Plexiglas box (35 × 20 × 14 cm) and observed for the presence or absence of maximal (tonic) hindlimb extension.

The threshold for maximal seizures was determined according to the above-mentioned method described by Kimball et al. [23]. Each mouse was stimulated only once at any given current intensity. The data obtained in groups of 19–21 mice were used to determine the threshold current intensity which induces the hindlimb extension in 50% of the animal tested (CS_50_ in mA).

### The Timed *iv* PTZ Infusion Test in Mice

At the appropriate time after drugs suspensions or vehicle administration mice were placed in the restrainer and a needle (27G, 3/4 in., Sterican^®^, B. Braun Melsungen AG, Melsungen, Germany) was inserted in the lateral tail vein. The needle was connected by polyethylene tube (PE20RW, Plastics One Inc. Roanoke, VA, USA) with a plastic syringe that was placed in the syringe pump (model Physio 22, Hugo Sachs Elektronik-Harvard Apparatus GmbH, March-Hugstetten, Germany). The proper placement of the needle in the vein was confirmed by appearance of blood in the cannula and afterwards the needle was secured to the tail by adhesive tape. The syringe contained 1% solution of PTZ in saline, which was administered into the vein of unrestrained animal at a constant rate of 0.2 ml/min. The time intervals from the start of infusion of PTZ solution to the appearance of three separate endpoints, i.e., first myoclonic twitch, generalized clonus with loss of righting reflex and forelimb tonus, were recorded. The threshold, in mg of PTZ per kg of body weight, for each endpoint was calculated according to the following formula:

$${{\text{PTZ}}\left( {{\text{mg}}/{\text{kg}}} \right) = \frac{{\text{Infusion duration (s)} \times \text{infusion rate (ml/s)} \times \text {PTZ concentration (mg/ml)}}}{{\text{Weight (kg)}}}}$$

Each group consisted of 10–15 animals.

### Chimney Test and Grip-Strength Test

Chimney test [25] was used to detect the motor deficits in mice induced by BBG. In this test, the inability of animals to climb back-ward up through a Plexiglas tube (3 cm, inner diameter × 30 cm, length) within 60 s is an indicator of motor impairment. Each group consisted of 11–12 mice.

Influence of BBG on muscular strength in mice was determined in the grip-strength test [26]. The apparatus for this test consisted of a steel wire grid (8 × 8 cm) connected to an isometric force transducer. Mice were lifted by their tail so that they grasp the grid with their forepaws. The mice were then gently pulled backward until they released the grid and the maximal force in millinewtons (mN) exerted by the mouse before losing grip was measured. The mean of three consecutive measurements for each animal was calculated and normalized to body weight (mN/g). Each experimental group consisted of 11–12 animals.

The chimney test and the grip strength test are quick and non-invasive procedures, and thus they were performed in the same groups of animals before seizure tests. This made it possible to reduce the total number of animals used for experiments, which was in accordance with the recommendations of the Ethical Committee.

### Statistical Analysis

Seizure thresholds in the 6 Hz and MEST tests are expressed as CS_50_ values with their 95% confidence limits. CS_50_ values were compared with the one-way analysis of variance (ANOVA) followed by the post-hoc Tukey’s test for multiple comparisons. The results from the *iv* PTZ test are presented as mean threshold doses of PTZ in mg/kg (+SEM) which induce the first myoclonic twitch, generalized clonus with loss of righting reflex and forelimb tonus in mice. The differences between seizure thresholds in this test were analyzed with one-way ANOVA with Tukey’s post-hoc test. Moreover, differences between the groups treated with VPA (positive control) and the appropriate groups treated with saline (negative control) were analyzed using one-tailed Student’s *t* test.

Results from the chimney test are presented as the percent of mice with impaired coordination in a group and statistical analysis was performed with Fisher’s exact probability test. Statistical analysis of data from the grip-strength test was performed with one-way ANOVA.

Statistical significance was noted when p values were equal to or < 0.05.

## Results

### Time-Course and Dose–Response Relationship for BBG in the 6 Hz Seizure Test in Mice

**Fig. 1:**
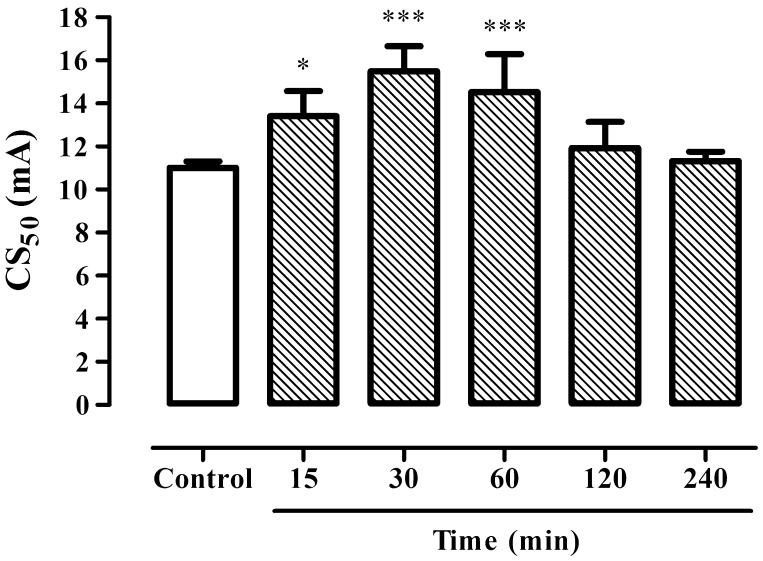
Time course effect of BBG on the threshold for 6 Hz-induced seizures in mice. Results are presented as median current strengths (CS_50_ in mA with their 95% confidence limits as the *error bars*) required to produce psychomotor seizures in 50% of animal tested. BBG at a dose of 200 mg/kg was administered *ip* 15, 30, 60, 120 and 240 min prior to the test. One-way ANOVA followed by the Tukey’s post-hoc multiple comparison test was used to analyze the data. *p < 0.05 and ***p < 0.001 versus control group

BBG at a dose of 200 mg/kg showed statistically significant anticonvulsant effect 15, 30 and 60 min after its acute administration in the 6 Hz seizure test in mice (Fig. 1; one-way ANOVA: F(5,50) = 13.06; p < 0.0001). Seizure threshold increased from 11.02 (10.73–11.31) mA in control group (saline treated) to 13.41 (12.33–14.58) mA 15 min after BBG administration (p < 0.05). The higher anticonvulsant effect was noted 30 and 60 min after drug treatment and seizure thresholds had a value of 15.49 (14.40–16.66) mA and 14.52 (12.94–16.29) mA, respectively (p < 0.001 vs. control group).

**Fig. 2:**
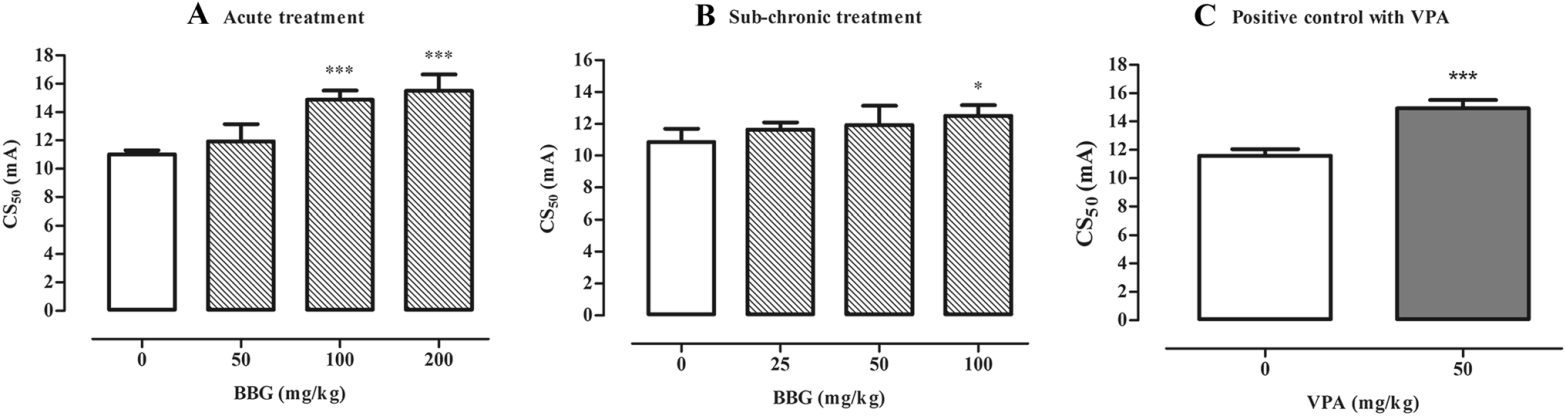
Effect of BBG on 6 Hz-induced psychomotor seizure threshold in mice. BBG was administered acutely 30 min prior to the test at doses ranging from 50 to 200 mg/kg (**a**) or sub-chronically for seven consecutive days at doses ranging from 25 to 100 mg/kg and the test was performed 30 min after the last injection (**b**). VPA (positive control) was injected 15 min before the test (acute treatment) at a dose of 50 mg/kg (**c**) and tested to verify reliability of the method. Results are presented as median current strengths (CS_50_ in mA with their 95% confidence limits as the *error bars*) required to produce psychomotor seizures in 50% of animal tested. One-way ANOVA followed by the Tukey’s post-hoc multiple comparison test was used to analyze the data received in the groups treated with BBG while effect of VPA was analyzed using Student’s *t* test. *p < 0.05 and ***p < 0.001 versus control group

BBG administered acutely at doses ranging from 50 to 200 mg/kg increased seizure thresholds in the 6 Hz test (Fig. 2a) although statistically significant changes were noted in groups which were treated with BBG at doses of 100 and 200 mg/kg (one-way ANOVA: F(3,32) = 26.64, p < 0.0001). In control group, seizure threshold was 11.02 (10.73–11.31) mA while in group of animals treated with BBG at a dose of 100 mg/kg it had a value of 14.88 (14.26–15.53) mA. BBG administered at a dose of 200 mg/kg raised seizure threshold to 15.49 (16.66–14.40) mA.

After sub-chronic administration of BBG at doses of 25–100 mg/kg, statistically significant increase in seizure threshold in the 6 Hz seizure test in mice was noted in a group of animals injected with BBG at a dose of 100 mg/kg (Fig. 2b; one-way ANOVA: F(3,34) = 2.976, p = 0.0452). In control group, seizure threshold was 10.87 (10.10–11.69) mA while after sub-chronic administration of BBG at a dose of 100 mg/kg raised to 12.50 (11.86–13.16) mA.

Moreover, VPA (positive control) administered *ip* 15 min before the 6 Hz test caused statistically significant increase in psychomotor seizure threshold from 11.57 (11.10–12.06) mA in control (saline-treated) group to 14.93 (14.35–15.53) mA (Fig. 2c, Student’s *t* test: p < 0.0001).

### Effect of BBG on Seizure Threshold in MEST and *iv* PTZ Tests in Mice

**Fig. 3:**
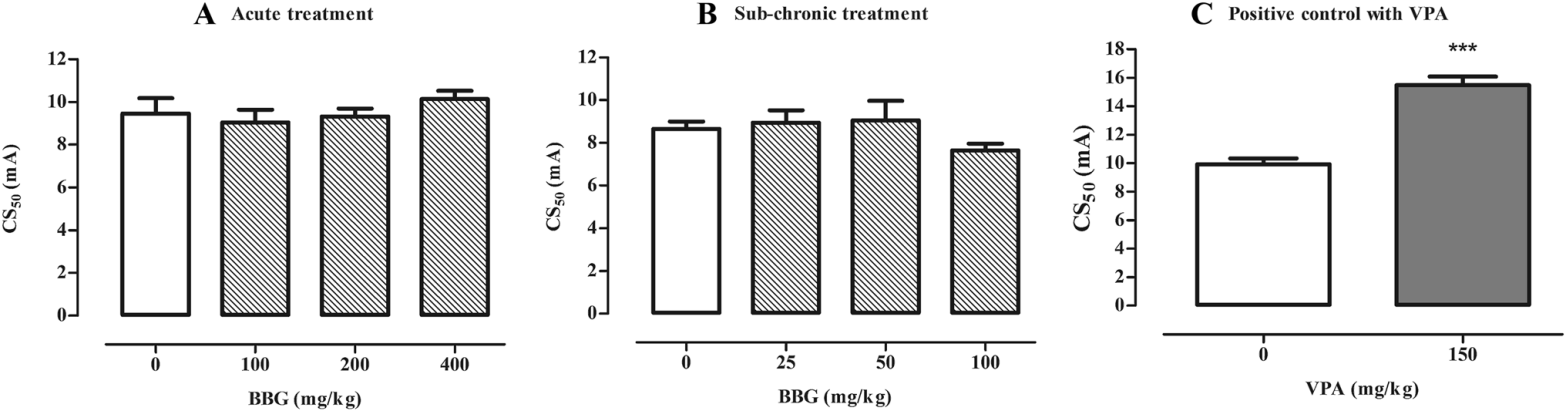
Effect of BBG on seizure threshold in the MEST test in mice. BBG was administered acutely 30 min prior to the test at doses ranging from 100 to 400 mg/kg (**a**) or sub-chronically for seven consecutive days at doses ranging from 25 to 100 mg/kg and the test was performed 30 min after the last injection (**b**). VPA (positive control) was injected 15 min before the test (acute treatment) at a dose of 150 mg/kg (**c**) and tested to verify reliability of the method. Results are presented as median current strengths (CS_50_ in mA with their 95% confidence limits as the *error bars*) required to produce tonic hindlimb extension in 50% of animal tested. One-way ANOVA followed by the Tukey’s post-hoc multiple comparison test was used to analyze the data received in the groups treated with BBG while effect of VPA was analyzed using Student’s *t* test. ***p < 0.001 versus control group

Neither acute (doses ranging of 100–400 mg/kg) nor sub-chronic (doses ranging of 25–100 mg/kg) administration of BBG caused any statistically significant changes in seizure thresholds in comparison to the respective control group in the MEST test in mice (Fig. 3a, b; acute treatment; one-way ANOVA: F(3,34) = 3.00, p = 0.044; sub-chronic treatment; one-way ANOVA: F(3,33) = 5.767, p = 0.0028). VPA (positive control) at a dose of 150 mg/kg increased seizure threshold in the MEST test by ~56% in comparison to control group (Fig. 3c, Student’s *t* test: p < 0.0001 vs. saline-treated group).

**Fig. 4:**
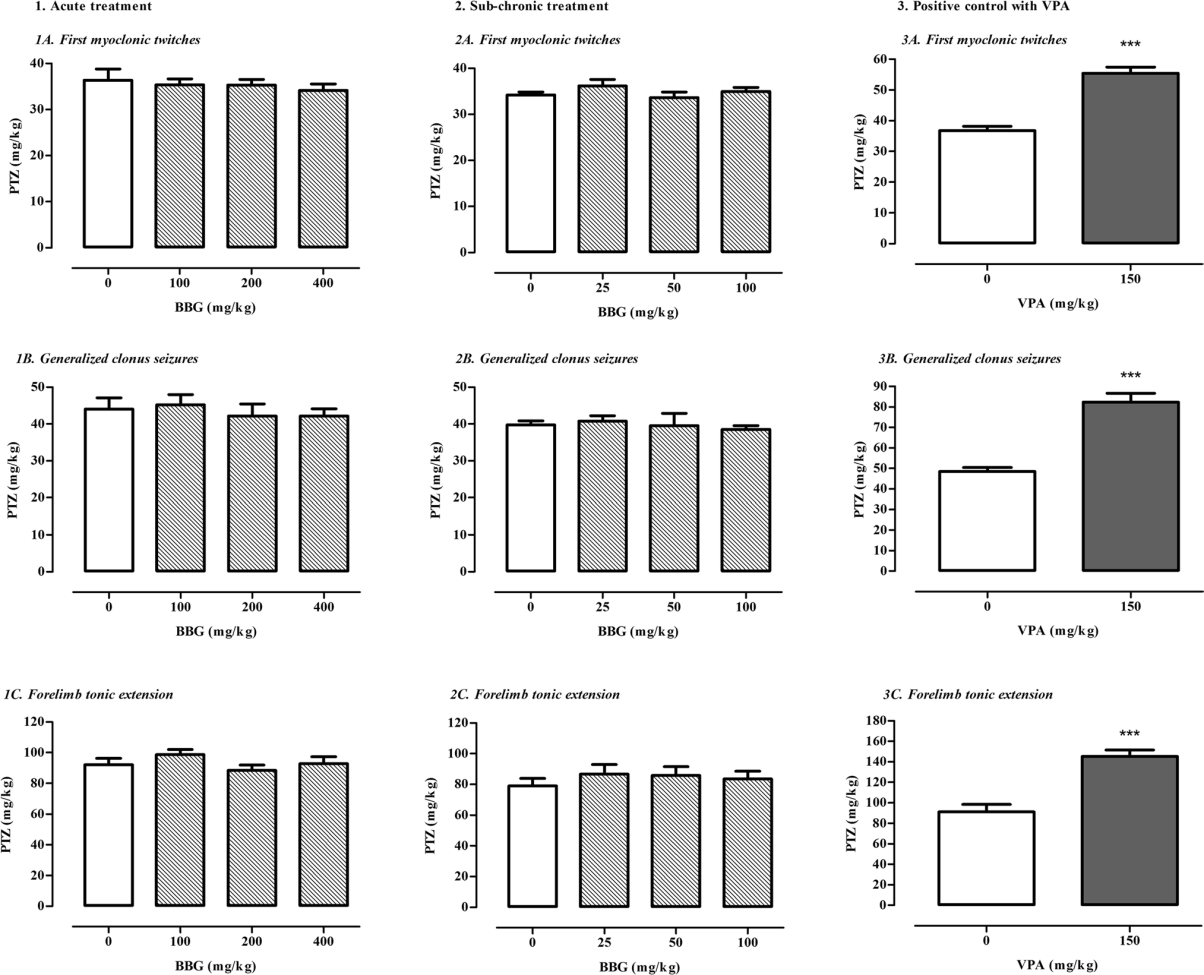
Effect of BBG on the thresholds for the first myoclonic twitch (**1a**, **2a**), generalized clonic seizures (**1b**, **2b**) and forelimb tonic extension (**1c**, **2c**) in the *iv* PTZ test in mice. BBG was administered acutely 30 min prior to the test at doses ranging from 100 to 400 mg/kg or sub-chronically for seven consecutive days at doses ranging from 25 to 100 mg/kg and the test was performed 30 min after the last injection. VPA (positive control) was injected 15 min before the test (acute treatment) at a dose of 150 mg/kg and tested to verify reliability of the method (**3a**, **3b**, **3c**). Data are presented as means + SEM. One-way ANOVA followed by the Tukey’s post-hoc multiple comparison test was used to analyze the data received in the groups treated with BBG while effect of VPA was analyzed using Student’s *t* test. ***p < 0.001 versus control group

BBG administered acutely (doses ranging of 100–400 mg/kg) and sub-chronically (doses ranging of 25–100 mg/kg) did not change significantly seizure thresholds for first myoclonic twitches (acute treatment; one-way ANOVA: F(3,35) = 0.2771, p = 0.8415; sub-chronic treatment; one-way ANOVA: F(3,54) = 1.047, p = 0.3793), generalized clonus seizures (acute treatment; one-way ANOVA: F(3,36) = 0.2699, p = 0.8467; sub-chronic treatment; one-way ANOVA: F(3,53) = 0.2714, p = 0.8457) and forelimb tonic extension (acute treatment; one-way ANOVA: F(3,32) = 1.064, p = 0.378; sub-chronic treatment; one-way ANOVA: F(3,52) = 0.4237, p = 0.7368) in the *iv* PTZ test in mice. VPA (at a dose of 150 mg/kg), which was tested as a positive control in this test, caused statistically significant increase in the thresholds for all three seizure kinds (Student’s *t* test: p < 0.0001 vs. saline-treated group). Data are shown in Fig. 4.

### Effect of BBG in the Grip Strength and Chimney Tests in Mice

BBG administered acutely (at doses of 100–400 mg/kg) and sub-chronically (at doses of 25–100 mg/kg) did not cause any statistically significant changes in muscular strength (Table 1) which was assessed in the grip strength test in mice (one-way ANOVA: acute treatment; F(3,43) = 0.116, p = 0.9503; sub-chronic treatment; F(3,44) = 1.810, p = 0.1593). Moreover, neither acute nor sub-chronic administration of BBG produced statistically significant changes in motor coordination in the chimney test in mice (Table 1, p > 0.05).

**Table 1:** Effects of BBG in the grip strength and chimney tests in mice

Treatment (mg/kg)	Neuromuscular strength (mN/g)	Impairment of motor performance (%)
I. Acute treatment		
Vehicle (control)	30.68 ± 1.02	16.6
VPA (150)	28.72 ± 2.11	8.3
II. Acute treatment		
Vehicle (control)	30.85 ± 0.93	9.1
BBG (100)	30.85 ± 0.74	0
BBG (200)	31.13 ± 1.14	0
BBG (400)	30.34 ± 1.03	36.36
III. Sub-chronic treatment		
Vehicle (control)	26.87 ± 1.06	0
BBG (25)	29.75 ± 0.92	0
BBG (50)	27.98 ± 0.99	0
BBG (100)	26.69 ± 1.19	0

Results are presented as mean (±SEM) grip strengths in millinewtons per gram of mouse body weight (mN/g) from the grip-strength test, assessing neuromuscular strength in mice, and as percentage of animals showing motor coordination impairment in chimney test in mice. Each experimental group consisted of 11–12 animals Statistical analysis of data from the grip-strength test was performed with *t* Student’s test (part I; p = 0.206) and one-way ANOVA (part II: F(3,43) = 0.116; p = 0.9503; part III: F(3,44) = 1.81; p = 0.1593) and the Fisher’s exact probability test (part I, II and III p > 0.05) was used to analyze the results from the chimney test

VPA administered acutely at a dose of 150 mg/kg also did not affect significantly muscular strength (Student’s *t* test: p > 0.05) and motor coordination (Fisher’s exact probability test: p > 0.05) in mice.

## Discussion

The aim of the present study was to investigate the anticonvulsant potential of BBG, a potent and selective antagonist of P2X7 receptor [21], in three well-established acute seizure models, i.e., in the 6 Hz-induced psychomotor seizures, MEST and *iv* PTZ tests in mice. Both the MEST and PTZ tests are experimental models of seizures which are considered as a “gold standard” in screening new substances with anticonvulsant activity. The MEST test is recognized as a model of tonic-clonic seizures in patients while the *iv* PTZ test is thought to be a model of generalized nonconvulsive seizures. The 6 Hz psychomotor seizure test has been added in recent years to procedures used by the Anticonvulsant Screening Program (ASP)/Epilepsy Therapy Screening Program (ETSP) at the University of Utah during searching new therapeutics for epileptic patients and it has been described as model of resistant epilepsy. However, Löscher et al. [27] have recently argued that 6 Hz induced seizures should not be considered as a model of drug-refractory partial seizures because these seizures seem to be refractory mainly to sodium channel modulators, e.g., phenytoin and lamotrigine, whereas drugs with other mechanism of action, especially GABAergic compounds, are quite effective in this test. Models which we used in the present study allow initial differentiation of the potential anticonvulsant effect of BBG and they are thought to be useful to identify anticonvulsant drugs that block different kinds of seizures in epileptic patients. Although the used seizure models are well-proven and widely used in screening substances with potential anticonvulsant effects, we investigated the effect of VPA in these tests as a positive control to additionally validate reliability of these methods. Our findings confirm the reliability and property of the used methods because classical antiepileptic drug, i.e., VPA, caused statistically significant increase in the seizure thresholds versus appropriate control (saline-treated) group in all cases analyzed.

In the last decade, there has been growing interest in relationship between P2X7 receptors and epileptogenic processes. Moreover, it is widely suggested that these purinergic receptors might be a new target in epilepsy treatment [5]. Firstly, numerous studies demonstrated increased expression of P2X7 receptor protein in brains of epileptic animals and patients with temporal lobe epilepsy. Dona et al. [28] reported heightened expression of these receptors both in glial cells and glutamatergic terminals in neocortex during acute and chronic phase of epilepsy in pilocarpine model in rats. This study did not revealed any significant changes in expression of hippocampal P2X2 and P2X4 receptors during acute and latent phase of epilepsy, which might suggest that these receptors are not so meaningful in epileptogenic processes [28]. Upregulation of the P2X7 receptor was also noted in hippocampus in pilocarpine model in rats [29, 30] and both in hippocampus and neocortex after status epilepticus induced by intra-amygdala microinjection of kainic acid in mice [6, 31, 32]. It was reported that P2X7 receptors are characteristic for astrocytes [7]. Loss and/or dysfunctions of these cells might play significant role in epileptogenesis and these processes were investigated in pilocarpine-induced model of status epilepticus in rats. Received results showed that BBG and OxATP (P2X7 receptor antagonists) prevented apoptotic loss of astrocytes in molecular layer of the dentale gyrus and frontoparietal cortex in these animals [33].

Participation of P2X7 receptors in epileptogenesis was also confirmed in clinical studies. Higher expression of these purinergic receptors was noted in neocortical neurons in patients with epilepsy [32, 34]. Moreover, it is supposed that P2X7 receptors participate in development of pharmacologically resistant epilepsy in patients with focal cortical dysplasia. Their increased expression was noted in some abnormal cells characteristic for this disorder, i.e., in dysmorphic cells and balloon cells. Moreover overexpression of P2X7 receptor in surgically resected cortical lesions of these patients was connected with higher expression of interleukin β1, a downstream factor of the P2X7 receptor signaling pathway [35].

There are also some studies which demonstrate anticonvulsant and antiepileptogenic properties of P2X7 receptor antagonists in some animal models of epilepsy. BBG and A-438079 markedly weakened both electrographic and behavioral seizures during status epilepticus triggered by intra-amygdala microinjection of kainic acid in mice [6, 32]. Another P2X7 receptor antagonist, i.e., JNJ-42253432, significantly reduced the severity of spontaneously recurrent seizures but did not affect their frequency in the established chronic epilepsy phase after kainic acid administration in rats [36]. Injection of kainic acid into the amygdala in mice raised expression of P2X7 receptor protein in CA1 pyramidal and granule neurons, as well as in microglia. Systemically injected JNJ-47965567 (the centrally available potent and specific P2X7 receptor antagonist) reduced both spontaneous seizures monitored in EEG recording and restricted mioglosis and astrogliosis processes in hippocampus in this model [31]. In addition, other studies revealed that BzATP, a P2X7R agonist, potentiated the electrographic seizures in mice with kainic acid-induced status epilepticus [6, 32]. Fischer et al. [37] reported that some P2X7 receptor antagonists, i.e., BBG, AFC-5128, JNJ-47965567 and tanshinone IIA sulfonate, attenuate PTZ-induced kindling development in rats by reduction of mean seizure stage. Moreover, in study conducted by Soni et al. [38] BBG not only decreases the mean score of PTZ-kindled seizures but also prevented associated cognitive and motor coordination impairments. This effect of P2X7 receptor antagonist was potentiated by ceftriaxone—a GLT-1 upregulator [38]. A740003, a P2X7 receptor antagonist, reduced the amplitude of slow field potentials of recurrent epileptiform discharges induced by high extracellular potassium concentration combined with application of pilocarpine in naïve rats but did not significantly affect these parameters in pilocarpine-treated chronic epileptic rats [7].

Activity of BBG and three other CNS-permeable P2X7 receptors blockers, i.e., AFC-5128, JNJ-47965567 and tanshinone, has been recently studied in two acute seizure models in mice, i.e., in the MEST and subcutaneous (*sc*) PTZ tests [37]. In our research, we used the additional seizure model, i.e., the 6 Hz-induced psychomotor seizure test in mice, and we also replaced the *sc* PTZ test with the *iv* PTZ test which is thought to be more sensitive and allow to determine the effect of the studied compound on thresholds for three separate seizure endpoints, i.e., the first myoclonic twitch, generalized clonus with loss of righting reflex and forelimb tonus. Furthermore, this test is appropriable to detect proconvulsant potential of the studied substance [39]. In addition to acute administration of BBG, we used also sub-chronic treatment with BBG. BBG was administered once, 30 min before the respective seizure test, to check effect of acute blockade of P2X7 receptor, and sub-chronically by seven consecutive days, to check whether prolonged inhibition of P2X7 receptors influences seizure activity in the used experimental models.

Our results revealed only slight anticonvulsant action of BBG because statistically significant increase in the seizure threshold was noted only in the 6 Hz test in mice. BBG did not affect seizure thresholds in the MEST and *iv* PTZ tests in mice, which is consistent with the results presented by Fischer et al. [37]. They ascertained that studied P2X7 receptor antagonists change neither threshold for tonic hindlimb extension in the MEST test nor latency to generalized clonic seizures in the *sc* PTZ test [37]. Moreover, our study revealed that sub-chronic administration of BBG did not improve its anticonvulsant activity in the used acute seizure models in mice. Based on the above mentioned results, it might be postulated that inhibition of P2X7 receptors does not play significant role in inhibition of seizures.

Results of many studies show that anticonvulsant and antiepileptic activity of some P2X7 receptor antagonists observed in animal models of epilepsy (i.e., kindling model, kainic acid and pilocarpine models) might arise from their anti-inflammatory action [20, 35]. The lack of anticonvulsant action of BBG in the present study may not be surprising, as there is no inflammation in used acute seizure models. In the present study, convulsions were triggered in healthy animals as a result of electrical stimulation (MEST and 6 Hz tests) or administration of chemoconvulsant—PTZ. In these models, inflammatory processes are not relevant and thus BBG, a selective P2X7 receptor antagonist, could not exhibit its anti-inflammatory and potentially anticonvulsant effect. There are also other compounds with anti-inflammatory properties which revealed protective action in models of epilepsy but did not affect seizure activity in acute seizure models in mice. So like luteolin, it alleviated the seizure score, elevation of glutamate levels in the hippocampus, neuronal death and microglial activation in hippocampus after kainic acid administration in rats [40] but it did not affect seizures in the 6 Hz and MEST tests in mice [41]. Although rutin and quercetin showed diverse protective effects in some epilepsy models [42–45] they had limited influence on the seizure thresholds in acute seizure models. They increased seizure threshold only in the 6 Hz test and were ineffective in the MEST and *iv* PTZ tests in mice [46].

Difference in activity of BBG in the used seizure models might also result from influence of the studied compound on the specific brain regions which are involved in seizure activity in these tests. Barton et al. [22] used the immediate early gene c-Fos as a marker of seizure-induced neuronal activation to determine structures which are involved in convulsant activity in the 6 Hz, *iv* PTZ and MES tests. They revealed that 6 Hz simulation with low current intensity, i.e., 22 and 32 mA, does not significantly activate hippocampus regions while they are highly active both after 6 Hz simulation with high intensity, i.e., 44 mA, and in the MES and PTZ tests. In our study we did not used high current intensity stimulation in the 6 Hz test and therefore we could suppose that there were no significant activation of hippocampus. It is probable that lack of anticonvulsant activity of BBG in the MEST and *iv* PTZ is caused by high activity of hippocampus regions.

There is also evidence that P2X7 receptor-mediated current increases Ca^2+^ entry into neuron cells and thereby might influence excitability of neurons. This fact and presynaptic localization of these receptors cause that they might influence neurotransmitters release and thereby control excitatory processes in neuronal cells [20]. However, it might be presumed that influence of P2X7 receptors on neuronal excitability is too subtle to modulate significantly seizure threshold in acute seizure models.

Although BBG is considered as a selective antagonist of P2X7 receptor, there is probability that at high doses (which were used in our study) it might affect other purinergic receptors, mainly P2X2 and P2X4 [21]. Participation of these two kinds of purinergic receptors in seizures and epileptogenic processes have been less investigated but seems to be much less important than P2X7 receptors [20]. Nevertheless, we cannot exclude interactions with other types of receptors, which could affect seizure susceptibility in the used seizure tests.

## Conclusions

Although some P2X7 receptor antagonists have been reported to produce potent anticonvulsant effects in different animal models of status epilepticus, ability of BBG, a selective antagonist of P2X7 receptor, to suppress acute seizures in animal models is very limited. Both contribution of these receptors in epileptogenic processes and anticonvulsant properties of their antagonists should be further investigated.

## Abbreviations

AEDs: Antiepileptic drugs
ATP: Adenosine 5′-triphosphate
BBG: Brilliant blue G
*ip*: Intraperitoneally
*iv*: Intravenously
MEST: Maximal electroshock seizure threshold
PTZ: Pentylenetetrazole
*sc*: Subcutaneously
SEM: Standard error of the mean

## References

[1] Engel T, Alves M, Sheedy C, Henshall DC (2016). ATPergic signalling during seizures and epilepsy. Neuropharmacology.

[2] Galanopoulou AS, Buckmaster PS, Staley KJ, Moshé SL, Perucca E, Engel J Jr, Löscher W, Noebels JL, Pitkänen A, Stables J, White HS, O’Brien TJ, Simonato M (2012) Identification of new epilepsy treatments: issues in preclinical methodology. Epilepsia 53:571–582 10.1111/j.1528-1167.2011.03391.x PMC3551973 22292566

[3] Pitkänen A, Löscher W, Vezzani A, Becker AJ, Simonato M, Lukasiuk K, Gröhn O, Bankstahl JP, Friedman A, Aronica E, Gorter JA, Ravizza T, Sisodiya SM, Kokaia M, Beck H (2016) Advances in the development of biomarkers for epilepsy. Lancet Neurol 15:843–856 10.1016/S1474-4422(16)00112-5 27302363

[4] Diaz-Hernández M, Diez-Zaera M, Sánchez-Nogueiro J, Gómez-Villafuertes R, Canals JM, Alberch J, Miras-Portugal MT, Lucas JJ (2009) Altered P2X7-receptor level and function in mouse models of Huntington’s disease and therapeutic efficacy of antagonist administration. FASEB J 23:1893–1906 10.1096/fj.08-122275 19171786

[5] Engel T, Jimenez-Pacheco A, Miras-Portugal MT, Diaz-Hernandez M, Henshall DC (2012). P2X7 receptor in epilepsy; role in pathophysiology and potential targeting for seizure control. Int J Physiol Pathophysiol Pharmacol.

[6] Engel T, Gomez-Villafuertes R, Tanaka K, Mesuret G, Sanz-Rodriguez A, Garcia-Huerta P, Miras-Portugal MT, Henshall DC, Diaz-Hernandez M (2012). Seizure suppression and neuroprotection by targeting the purinergic P2X7 receptor during status epilepticus in mice. FASEB J.

[7] Klaft ZJ, Schulz SB, Maslarova A, Gabriel S, Heinemann U, Gerevich Z (2012). Extracellular ATP differentially affects epileptiform activity via purinergic P2X7 and adenosine A1 receptors in naive and chronic epileptic rats. Epilepsia.

[8] Skaper SD, Debetto P, Giusti P (2010). The P2X7 purinergic receptor: from physiology to neurological disorders. FASEB J.

[9] Sperlagh B, Illes P (2014). P2X7 receptor: an emerging target in central nervous system diseases. Trends Pharmacol Sci.

[10] Diaz-Hernandez JI, Gomez-Villafuertes R, León-Otegui M, Hontecillas-Prieto L, Del PA, Trejo JL, Lucas JJ, Garrido JJ, Gualix J, Miras-Portugal MT, Diaz-Hernandez M (2012) In vivo P2X7 inhibition reduces amyloid plaques in Alzheimer’s disease through GSK3beta and secretases. Neurobiol Aging 33:1816–1828 10.1016/j.neurobiolaging.2011.09.040 22048123

[11] Goloncser F, Sperlagh B (2014). Effect of genetic deletion and pharmacological antagonism of P2X7 receptors in a mouse animal model of migraine. J Headache Pain.

[12] Gum RJ, Wakefield B, Jarvis MF (2012). P2X receptor antagonists for pain management: examination of binding and physicochemical properties. Purinergic Signal.

[13] Sperlagh B, Csolle C, Ando RD, Goloncser F, Kittel A, Baranyi M (2012). The role of purinergic signaling in depressive disorders. Neuropsychopharmacol Hung.

[14] Stokes L, Spencer SJ, Jenkins TA (2015). Understanding the role of P2X7 in affective disorders-are glial cells the major players?. Front Cell Neurosci.

[15] Gever JR, Cockayne DA, Dillon MP, Burnstock G, Ford AP (2006). Pharmacology of P2X channels. Pflugers Arch.

[16] Strauss KI, Elisevich KV (2016). Brain region and epilepsy-associated differences in inflammatory mediator levels in medically refractory mesial temporal lobe epilepsy. J Neuroinflammation.

[17] Mesuret G, Engel T, Hessel EV, Sanz-Rodriguez A, Jimenez-Pacheco A, Miras-Portugal MT, Diaz-Hernandez M, Henshall DC (2014). P2X7 receptor inhibition interrupts the progression of seizures in immature rats and reduces hippocampal damage. CNS Neurosci Ther.

[18] Vieira V, Glassmann D, Marafon P, Pereira P, Gomez R, Coitinho AS (2016). Effect of diclofenac sodium on seizures and inflammatory profile induced by kindling seizure model. Epilepsy Res.

[19] Cieślak M, Wojtczak A, Komoszyński M (2016). Role of the purinergic signaling in epilepsy. Pharmacol Rep.

[20] Henshall DC, Diaz-Hernandez M, Miras-Portugal MT, Engel T (2013). P2X receptors as targets for the treatment of status epilepticus. Front Cell Neurosci.

[21] Jiang LH, Mackenzie AB, North RA, Surprenant A (2000). Brilliant blue G selectively blocks ATP-gated rat P2X(7) receptors. Mol Pharmacol.

[22] Barton ME, Klein BD, Wolf HH, White HS (2001). Pharmacological characterization of the 6Hz psychomotor seizure model of partial epilepsy. Epilepsy Res.

[23] Kimball AW, Burnett WT, Doherty DG (1957). Chemical protection against ionizing radiation. I. Sampling methods for screening compounds in radiation protection studies with mice. Radiat Res.

[24] Nieoczym D, Socała K, Jedziniak P, Olejnik M, Wlaź P (2013). Effect of sildenafil, a selective phosphodiesterase 5 inhibitor, on the anticonvulsant action of some antiepileptic drugs in the mouse 6-Hz psychomotor seizure model. Prog Neuropsychopharmacol Biol Psychiatry.

[25] Boissier JR, Tardy J, Diverres JC (1960). Une nouvelle méthode simple pour explorer l’action «tranquillisante»: le test de la cheminée. Med Exp (Basel).

[26] Meyer OA, Tilson HA, Byrd WC, Riley MT (1979). A method for the routine assessment of fore- and hindlimb grip strength of rats and mice. Neurobehav Toxicol.

[27] Löscher W (2016). The search for new screening models of pharmacoresistant epilepsy: is induction of acute seizures in epileptic rodents a suitable approach?. Neurochem Res.

[28] Dona F, Ulrich H, Persike DS, Conceicao IM, Blini JP, Cavalheiro EA, Fernandes MJ (2009). Alteration of purinergic P2X4 and P2X7 receptor expression in rats with temporal-lobe epilepsy induced by pilocarpine. Epilepsy Res.

[29] Vianna EP, Ferreira AT, Naffah-Mazzacoratti MG, Sanabria ER, Funke M, Cavalheiro EA, Fernandes MJ (2002). Evidence that ATP participates in the pathophysiology of pilocarpine-induced temporal lobe epilepsy: fluorimetric, immunohistochemical, and Western blot studies. Epilepsia.

[30] Da Silva Fernandes MJ, da Graça Naffah Mazzacoratti M, Cavalheiro EA (2010). Pathophysiological aspects of temporal lobe epilepsy and the role of P2X receptors. Open Neurosci J.

[31] Jimenez-Pacheco A, Diaz-Hernandez M, Arribas-Blázquez M, Sanz-Rodriguez A, Olivos-Oré LA, Artalejo AR, Alves M, Letavic M, Miras-Portugal MT, Conroy RM, Delanty N, Farrell MA, O’Brien DF, Bhattacharya A, Engel T, Henshall DC (2016) Transient P2X7 receptor antagonism produces lasting reductions in spontaneous seizures and gliosis in experimental temporal lobe epilepsy. J Neurosci 36:5920–5932 10.1523/JNEUROSCI.4009-15.2016 PMC6601816 27251615

[32] Jimenez-Pacheco A, Mesuret G, Sanz-Rodriguez A, Tanaka K, Mooney C, Conroy R, Miras-Portugal MT, Diaz-Hernandez M, Henshall DC, Engel T (2013). Increased neocortical expression of the P2X7 receptor after status epilepticus and anticonvulsant effect of P2X7 receptor antagonist A-438079. Epilepsia.

[33] Kim JE, Ryu HJ, Yeo SI, Kang TC (2011). P2X7 receptor differentially modulates astroglial apoptosis and clasmatodendrosis in the rat brain following status epilepticus. Hippocampus.

[34] Barros-Barbosa AR, Fonseca AL, Guerra-Gomes S, Ferreirinha F, Santos A, Rangel R, Lobo MG, Correia-de-Sa P, Cordeiro JM (2016). Up-regulation of P2X7 receptor-mediated inhibition of GABA uptake by nerve terminals of the human epileptic neocortex. Epilepsia.

[35] Wei YJ, Guo W, Sun FJ, Fu WL, Zheng DH, Chen X, Li S, Zang ZL, Zhang CQ, Liu SY, Yang H (2016). Increased expression and cellular localization of P2X7R in cortical lesions of patients with focal cortical dysplasia. J Neuropathol Exp Neurol.

[36] Amhaoul H, Ali I, Mola M, Van EA, Szewczyk K, Missault S, Bielen K, Kumar-Singh S, Rech J, Lord B, Ceusters M, Bhattacharya A, Dedeurwaerdere S (2016). P2X7 receptor antagonism reduces the severity of spontaneous seizures in a chronic model of temporal lobe epilepsy. Neuropharmacology.

[37] Fischer W, Franke H, Krugel U, Muller H, Dinkel K, Lord B, Letavic MA, Henshall DC, Engel T (2016). Critical evaluation of P2X7 receptor antagonists in selected seizure models. PLoS ONE.

[38] Soni N, Koushal P, Reddy BV, Deshmukh R, Kumar P (2015). Effect of GLT-1 modulator and P2X7 antagonists alone and in combination in the kindling model of epilepsy in rats. Epilepsy Behav.

[39] Mandhane SN, Aavula K, Rajamannar T (2007). Timed pentylenetetrazol infusion test: a comparative analysis with s.c.PTZ and MES models of anticonvulsant screening in mice. Seizure.

[40] Lin TY, Lu CW, Wang SJ (2016). Luteolin protects the hippocampus against neuron impairments induced by kainic acid in rats. Neurotoxicology.

[41] Shaikh MF, Tan KN, Borges K (2013). Anticonvulsant screening of luteolin in four mouse seizure models. Neurosci Lett.

[42] Baluchnejadmojarad T, Roghani M, Homayounfar H (2010). Inhibitory effect of high dose of the flavonoid quercetin on amygdala electrical kindling in rats. Basic Clin Neuroscience.

[43] Hu K, Li SY, Xiao B, Bi FF, Lu XQ, Wu XM (2011). Protective effects of quercetin against status epilepticus induced hippocampal neuronal injury in rats: involvement of X-linked inhibitor of apoptosis protein. Acta Neurol Belg.

[44] Nassiri-Asl M, Moghbelinejad S, Abbasi E, Yonesi F, Haghighi MR, Lotfizadeh M, Bazahang P (2013). Effects of quercetin on oxidative stress and memory retrieval in kindled rats. Epilepsy Behav.

[45] Nassiri-Asl M, Naserpour FT, Abbasi E, Sadeghnia HR, Sheikhi M, Lotfizadeh M, Bazahang P (2013). Effects of rutin on oxidative stress in mice with kainic acid-induced seizure. J Integr Med.

[46] Nieoczym D, Socała K, Raszewski G, Wlaź P (2014). Effect of quercetin and rutin in some acute seizure models in mice. Prog Neuropsychopharmacol Biol Psychiatry.

